# Forecasting age-standardized incidence rates of gastric cancer from 1990–2050 in Japan according to *H. pylori* prevalence and eradication scenarios

**DOI:** 10.1007/s00535-025-02296-y

**Published:** 2025-09-19

**Authors:** Byron Sigel, Eiko Saito, Daisuke Yoneoka, Tomohiro Matsuda, Kota Katanoda

**Affiliations:** 1 Department of Medicine, Washington University School of Medicine, St. Louis, MO USA; 2 Sustainable Society Design Center, Graduate School of Frontier Sciences, the University of Tokyo, 5-1-5 Kashiwanoha, Kashiwa-shi, Chiba, Japan; 3 National Institute of Infectious Diseases, Japan Institute for Health Security, Tokyo, Japan; 4 Center for Cancer Registries, National Cancer Center, Institute for Cancer Control, 5-1-1 Tsukiji, Chuo-ku, Tokyo, Japan; 5 Division of Population Data Science, National Cancer Center, Institute for Cancer Control, 5-1-1 Tsukiji, Chuo-ku, Tokyo, Japan

**Keywords:** *Helicobacter pylori*, Helicobacter eradication therapy, Gastric cancer incidence, Forecasting, Primary prevention

## Abstract

**Background:**

This study examines the influence of *H. pylori* eradication policies on gastric cancer incidence rates in Japan utilizing nationally representative registry data. It evaluates the impact of the *H. pylori* eradication policies introduced in 2000 and 2013, along with future eradication scenarios, on age-standardized gastric cancer rates.

**Methods:**

Data from prefectural cancer registries and national health surveys were analyzed using Poisson regression and autoregressive integrated moving average models. Predictors such as *H. pylori* prevalence, alcohol consumption, salt intake, body mass index, and smoking prevalence were included. The study assessed past policies by comparing incidence rates with and without the policy changes of 2000 and 2013. Future policies were evaluated through five scenarios, incorporating the cumulative impact of eradication efforts from 2000 and 2013, and a projected 75% reduction by 2050. The evaluation also compared eradication targets for age groups 40–69 and 20–39.

**Results:**

Past *H. pylori* eradication policies were associated with decreased age-standardized gastric cancer incidence rates in Japan, reducing the rate from a projected 39.3 per 100,000 without the 2000 and 2013 policies to 24.9 per 100,000 under current policies. Future policies, integrating the cumulative effects of the 2000 and 2013 eradication efforts and projecting a 75% reduction in *H. pylori* prevalence, were projected to further reduce gastric cancer incidence.

**Conclusion:**

The *H. pylori* eradication policies of 2000 and 2013 have significantly reduced gastric cancer incidence rates in Japan. Model projections suggest that expanded eradication efforts could lead to additional reductions, further lowering the future burden of gastric cancer in Japan.

**Supplementary Information:**

The online version contains supplementary material available at 10.1007/s00535-025-02296-y.

## Introduction

Globally, gastric cancer ranks as the fifth most prevalent cancer [1]. The incidence of gastric cancer is exceptionally high in East Asia, including Japan, where it was the third most prevalent type of cancer in 2019, with a fourfold higher incidence rate compared to the United Kingdom [1].

Several East Asian countries have initiated preventive programs to combat gastric cancer effectively. Early screening has been a crucial strategy in lowering the incidence of this disease. South Korea, for example, has seen a notable decrease in gastric cancer rates thanks to a comprehensive national screening program for citizens aged 40 and above [2, 3]. Similarly, Japan offers government-funded support for gastric cancer screening and treatment, underlining the critical role of early detection in improving health outcomes [4].

Recently, gastric cancer incidence and mortality have declined steadily in Japan. Katanoda, Ito and Sobue (2021) highlighted rapid declines in gastric cancer mortality from 1980 to 2016 [5, 6]. In Japan, several factors have contributed to the decrease in gastric cancer, including improved hygiene practices and a notable reduction in *Helicobacter pylori* infections [7–9]. *Helicobacter pylori* infection is acknowledged as a substantial risk factor for severe gastritis-associated diseases, such as peptic ulcers and gastric cancer [10–12].

Given the high prevalence of *H. pylori* infection in Japan, the country has instituted measures, such as subsidizing *H. pylori* eradication therapy [13]. The adoption of *H. pylori* eradication therapy has escalated over time; in 2000, the Japanese National Health Insurance (NHI) scheme sanctioned coverage of this therapy for peptic ulcers and expanded its approval for chronic gastritis (specifically, *H. pylori* gastritis) in 2013, reflecting a concentrated effort to mitigate the impact of such infections on public health [13–15].

Hiroi et al. [17] demonstrated a decrease in *H. pylori* prevalence in Japan with increased coverage for eradication therapy. Nevertheless, the study did not investigate the potential impact of this eradication therapy policy on gastric cancer incidence. While randomized controlled trials have demonstrated that *H. pylori* eradication therapy is effective in eradicating *H. pylori*, literature offers limited evidence concerning its policy implications, particularly regarding its impact on the age-standardized incidence of gastric cancer at a population level in Japan. In addition, it is unclear whether such therapy is effective in treating individuals who are asymptomatic or in populations that are younger. For instance, while *H. pylori* eradication therapy has been implemented in middle schools, the policy effects remain unclear [16].

Thus, we aim to evaluate the past policy effects of *H. pylori* eradication therapy in 2000 or 2013 on gastric cancer incidence rates in Japan. In addition, we predict the future impact of different eradication policies on gastric cancer incidence rates, based on various policy scenarios and targets.

## Methods

### Target population

This study evaluates the policy effects of *H. pylori* eradication therapy on gastric cancer incidence among adults aged 20–69 in Japan. The observed data included cases diagnosed with gastric cancer between January 1 st, 1990, and December 31 st, 2015.

### Data sources

#### Gastric cancer incidence

We used population-based cancer registry data from three high-quality prefectures (Yamagata, Fukui, Nagasaki) in the Monitoring of Cancer Incidence in Japan (MCIJ). Gastric cancers were identified based on the International Classification of Diseases for Oncology Third Edition (ICD-O-3) morphology code of C16.

#### Population-level predictors data including H. pylori infection

Our model for gastric cancer incidence includes five predictors, *H. pylori* prevalence, alongside smoking, alcohol consumption, salt intake, and BMI. *H. pylori* prevalence was based on a systematic review and meta-regression analysis by Wang et al. [17, 18]. The majority of the studies were cross-sectional and conducted as part of health screening programs, outpatient clinics, or community settings. To estimate the *H. pylori* prevalence in the past policy scenarios, we combined data from Wang et al. and Hiroi et al. [17, 19]. Wang et al. conducted a meta-regression of 46 studies (*n* = 170,752), providing birth-cohort- and age-specific prevalence estimates. To align with calendar-year-based gastric cancer incidence, we converted birth-year-specific estimates into calendar-year-specific values by applying age-stratified *H. pylori* prevalence rates to population distributions, using the 1985 Japanese standard population from the Statistics Bureau of Japan [20]. This approach is methodologically justified by the well-established natural history of *H. pylori* infection as acquisition typically occurs in early childhood and persists throughout adulthood unless eradicated via treatment [21–23]. Spontaneous clearance is rare, and reinfection rates in adults are low in developed settings such as Japan. Therefore, this static-cohort approach is appropriate for estimating *H. pylori* prevalence in Japan, where infection is typically acquired in early childhood, persists without treatment, and adult reinfection rates are low. Under these conditions, prevalence within a birth cohort can be reasonably assumed to remain stable over time. By incorporating changes in eradication uptake associated with policy shifts, this method allows projection of infection burden across calendar years without modeling dynamic transmission.

In addition, Hiroi et al. utilized the JMDC and MDV claims databases to identify patients receiving first-line *H. pylori* eradication therapy, validating treatment estimates with national sales and urea breath test data [19]. These inputs informed a Markov simulation model incorporating age-specific prevalence and demographic projections to estimate national infection trends through 2050 under varying policy scenarios. Their findings demonstrated an approximately 25% reduction in *H. pylori* prevalence by 2030 with a 2000 policy change alone, and a 50% reduction with both the 2000 and 2013 changes, compared to no policy change. Accordingly, we applied these relative reductions in our policy scenarios P1 (no policy change in 2000 and 2013) P2 (no policy change in 2013), and P3 (current policy).

To adjust for key behavioral and physiological factors in the model, we incorporated data on alcohol consumption, salt intake, BMI, and smoking prevalence from nationally representative surveys. Alcohol (percent alcohol consumption), salt intake (g/day), and BMI were obtained from the nationally representative household survey, Japan National Health and Nutrition Survey (JNHNS), from 1990 to 2012. Spline interpolation was used to impute the missing values. We calculated sodium intake in milligrams by multiplying by 2.54 and dividing by 1000. According to the Japan Society for the Study of Obesity, obesity is defined as having a body mass index (BMI) of 25 kg/m^2^ or higher [17, 24]. Smoking prevalence data were obtained from the Japan Tobacco (JT) Smoking Survey conducted between 1990 and 2012 [20, 25]. The JT survey uses a two-stage sampling method by mailing questionnaires to individuals aged 20 years or older [20, 25]. Given that only sex-specific rates were available, we calculated the overall smoking percentage using Japan’s annual country-level sex weights.

### Statistical model

Utilizing an ARIMA (autoregressive integrated moving average) model, we forecasted predictors, including *H. pylori* prevalence, alcohol, salt intake (g/day), BMI and smoking from 2013 to 2050. Moreover, independent predictor values were projected at the population level, using historical data (1990–2012) to forecast values from 2013 to 2050. Stationarity was confirmed using KPSS (Kwiatkowski–Phillips–Schmidt–Shin) tests, and non-stationary data were transformed as needed. Model parameters, which correspond to (p, d, q) in the following equation, were selected based on Akaike Information Criteria corrected for small sample size (AICc).

Subsequently, an ARIMAX (autoregressive integrated moving average with exogenous variables) model was employed to forecast log-scaled age-standardized gastric cancer incidence rates, incorporating forecasted predictors until 2050. The ARIMAX model is given by:

$$\left( {1 - \mathop \sum \limits_{i = 1}^{p} \alpha_{i} L^{i} } \right)(1 - L)^{d} y_{t} = \mathop \sum \limits_{j = 1}^{5} \gamma_{i} L^{d} x_{tj} + \left( {1 + \mathop \sum \limits_{i = 1}^{q} \beta_{i} L^{d} } \right) \varepsilon_{t} ,$$

where *x*_tj_ is the value of *j* th predictor at time *t*, γ_j_ is a regression coefficient parameter of the *j* th of 5 predictors, *y*_*t*_ represents log-scaled age-standardized rate of gastric cancer, *ε*_*t*_ denotes the white noise term at time *t*; *L* is the time lag operator defined as $L^{k} y_{t} = y_{t - k}$, and *α*_*i*_ and *β*_*i*_ are the ith coefficient parameters for the autoregressive (AR) component *p* and the moving average (MA) component *q*, respectively. Parameters were estimated separately across different age categories, with all analyses executed using R version 3.6.

To evaluate potential differences in the forecasted age-standardized incidence rates among scenarios, we examined the overlap in their 95% prediction intervals (PIs).

### Policy scenarios

#### Past policy scenarios

Table 1 (rows P1–P3) summarizes the evaluated past policy scenarios. Furthermore, scenarios assessing the impact of *H. pylori* eradication policies in 2000 and 2013 on gastric cancer incidence (2013–2050) were devised, grounded in Hiroi et al. 2017 [19]. Using the Japan Medical Data Center and Medical Data Vision databases, Hiroi et al. estimated future *H. pylori* prevalence, projecting a 14% *H. pylori* prevalence by 2030 with policy changes in 2000 and 2013, contrasting with higher rates if policies remained unaltered [18, 26]. Past policy scenarios 2 and 3 were compared based on the baseline scenario 1 as described below.

**Table 1:** Past and future policy scenarios and assumed effect sizes*

Scenarios†	Assumed *H. pylori* prevalence
*Past policy effects*	
P1: No policy change in 2000 and 2013	–
P2: No policy change in 2013	25% decrease from 2000 policy effect scenario
P3 Current policy scenario	50% decrease from 2000 policy effect scenario
*Future prediction*	
F1: Current policy scenario	See above
F2: Current policy + 2000 policy effect	25% decrease from current policy scenario
F3: Current policy + 2013 policy effect	33% decrease from current policy scenario
F4: Current policy + 2000 and 2013 policy effect	50% decrease from current policy scenario
F5: Current policy + 75% decrease	75% decrease from current policy scenario

*Scenarios were based on the estimations from Hiroi et al. 2017

†Percent decrease in *H. pylori* prevalence relative to 2030 rates

This study evaluated three scenarios related to these policies:

1. P1: Scenario with no policy change in 2000 and 2013, projecting the highest prevalence of *H. pylori* [19]
2. P2: Scenario with a policy change in 2000 but no policy change in 2013, leading to a 25% reduction in *H. pylori* prevalence in 2030 [19]
3. P3 (current policy scenario) with both the 2000 and 2013 policy changes, forecasting a 50% decrease in *H. pylori* prevalence in 2030 [19]

#### Future prediction by different eradication

Table 1 (rows F1–F5) summarizes the future policy scenarios. Specifically, we evaluated five future policy scenarios, extrapolating from past *Helicobacter pylori* eradication effects, to estimate the influence of increased *H. pylori* eradication on gastric cancer incidence rates. Furthermore, we also evaluated future policy scenarios for two different target age groups, particularly young adults aged 20–39 age group and older adults aged 40–69 age group. Scenarios 2, 3 4, and 5 were compared to scenario 1 as described below. *H. pylori* prevalence, relative to projected values for 2030:

1. F1 (current policy scenario) as described in scenario P3 of past policies above.
2. F2: 25% decrease in *H. pylori* prevalence corresponding to the same effect as 2000 insurance coverage expansion.
3. F3: 33% decrease in *H. pylori* prevalence corresponding to the same effect as the 2013 insurance coverage expansion.
4. F4: 50% decrease in *H. pylori* prevalence corresponding to the combined effect of both the 2000 and 2013 insurance coverage expansion.
5. F5: Best-case scenario expects a 75% reduction by 2030 in *H. pylori* prevalence.

We included the hypothetical best-case scenario, F5, to estimate the maximum potential impact of intensified *H. pylori* eradication on future gastric cancer incidence and to provide a benchmark for evaluating diminishing returns across more moderate intervention scenarios. For example, a mass eradication program in Taiwan achieved a 78.7% reduction in *H. pylori* prevalence over approximately 14 years, a timeframe comparable to the decline observed in our study from 2013 to 2030 [27]. Since sex-specific *H. pylori* prevalence data were unavailable, we utilized *H. pylori* prevalence data for both sexes in the model. However, sex-specific data were used for age-standardized gastric cancer incidence and other predictors.

#### Validation

To validate our model, we compared forecasted gastric cancer incidence rates based on current policy with the observed data from 2013 to 2015. Validation was performed for the 20–69 age group, as well as for the 20–39 and 40–69 age groups. We also examined the 95% PIs to assess the model’s reliability and applied external validation for the years for which we had observed data, specifically from 2013 to 2015, to determine the model’s accuracy in predicting gastric cancer incidence rates.

## Results

### Validation

The validation results for the ARIMAX model for 20–69 age group show that all data points, including observed values from 2013 to 2015, fall within the 95% PI (Supplementary Fig. S1). In addition, by target age group, there was a moderate underestimation for 20–39 age group, in which the observed gastric cancer incidence rates for 2014–2015 were higher than the model’s predictions (Supplementary Fig. S2). However, for the 40–69 age group, all the model’s predicted values for the analyzed years fell within the 95% PI (Supplementary Fig. S3). Furthermore, the age range of our target population (20–69 years) was similar to that used in Hiroi et al. 2017, which analyzed health insurance claims data with reported mean ages of 36.8 (JMDC) and 60.6 (MDV) [19]. Our estimated *H. pylori* prevalence was 14.7% in 2030 and 5.4% in 2050. These estimates closely align with Hiroi et al., who reported prevalence of 14% in 2030 and 5% in 2050, respectively.

### Evaluation of past policies

**Fig. 1:**
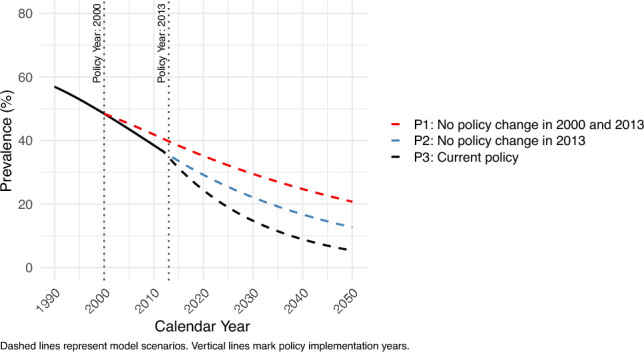
Projected *H. pylori* prevalence among individuals aged 20–69 years, both sexes, from 1990 to 2050 under three alternative past policy scenarios: P1 (no policy change in 2000 and 2013); P2 (no policy change in 2013); P3 (current policy scenario). The figure demonstrates that the implementation of more public health policies corresponds with larger projected declines in *H. pylori* prevalence over time

Figure 1 describes the *H. pylori* prevalence under past policy scenarios (P1–P3). The forecasted *H. pylori* prevalence is estimated as follows: P1 (no policy change in 2000 and 2013) is expected to have a prevalence of 29.5% by 2030; P2 (no policy change in 2013) with a prevalence of 22.1% by 2030; and P3 (current policy scenario) with a prevalence of 14.7% by 2030.

**Fig. 2:**
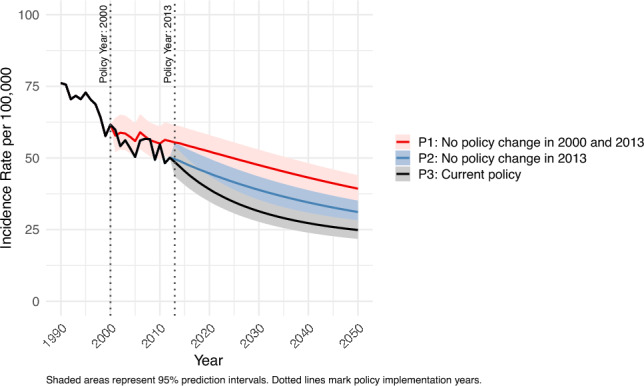
Projected age-standardized gastric cancer incidence rate among individuals aged 20–69 years, both sexes, from 1990 to 2050 under three alternative past policy scenarios: P1 (no policy changes in 2000 or 2013); P2 (no policy change in 2013); P3 (current policy scenario). The figure demonstrates that more comprehensive public health policies are associated with greater projected reductions in gastric cancer incidence over time

Based on these *H. pylori* prevalence scenarios and predictive factors, age-standardized incidence rates for each past policy scenario were projected from 2013 to 2050 (Fig. 2). We observed no remarkable difference in gastric cancer incidence rates between P3 (current policy scenario) and P2 (no policy change in 2013). However, after 2017, P3 demonstrated lower gastric cancer incidence rates compared to P1. Specifically, by 2050, the age-standardized incidence rate is projected to decrease to 24.9 per 100,000 under P3, compared to 39.3 per 100,000 under P1.

In addition, we forecasted age-standardized gastric cancer incidence by sex. Due to the absence of sex-specific *H. pylori* prevalence data, overall prevalence rates were applied uniformly to each sex. For males, the age-standardized incidence rates showed remarkable differences from 2023; the incidence rates were highest in P1 (no policy change in 2000 and 2013), followed by P2 (No policy change in 2013) and P3 (current policy scenario) (Supplementary Fig. S4). In contrast, for females, despite a noticeable difference between P3 and P1 from 2024 (Supplementary Fig. S5), the 95% PI for P2 overlapped with the other two scenarios.

### Future prediction by different eradication effects

**Fig. 3:**
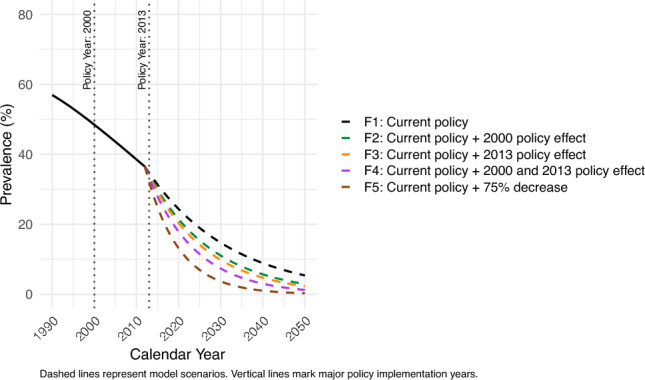
Projected *H. pylori* prevalence among individuals aged 20–69 years, both sexes, from 1990 to 2050 under five alternative future policy scenarios: F1 (current policy scenario); F2 (current policy + 2000 policy effect); F3 (current policy + 2013 policy effect); F4 (current policy + 2000 and 2013 policy effect); F5 (current policy + 75% decrease). The figure demonstrates that more intensive future interventions are associated with greater projected reductions in gastric cancer incidence

Figure 3 illustrates the forecasted *H. pylori* prevalence under future policy scenarios, with each scenario positing an increased *H. pylori* eradication. The forecasted *H. pylori* prevalence under future policy scenarios (in 2030) was highest in the following order: F1 (current policy scenario): 14.7%, F2 (current policy + 2000 policy effect): 11.1%, F3 (current policy + 2013 policy effect): 9.8%, F4 (current policy + 2000 and 2013 policy effect): 7.4%, and F5 (current policy + 75% decrease): 3.7%.

**Fig. 4:**
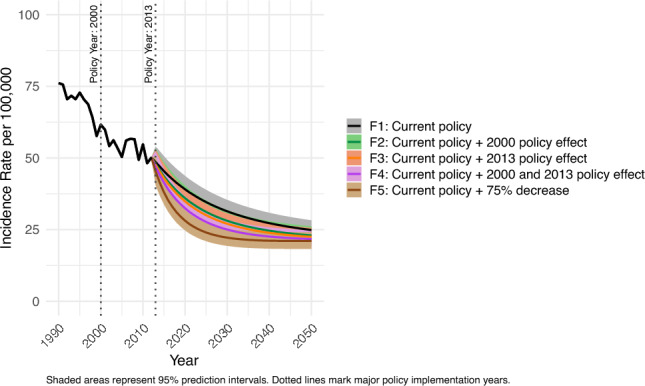
Projected age-standardized gastric cancer incidence rate among individuals aged 20–69 years, both sexes, from 1990 to 2050 under five alternative future policy scenarios: F1 (current policy scenario); F2 (current policy + 2000 policy effect); F3 (current policy + 2013 policy effect); F4 (current policy + 2000 and 2013 policy effect); F5 (current policy + 75% decrease). The figure demonstrates that more intensive future public health interventions are associated with greater projected reductions in gastric cancer incidence over time

Based on the scenarios for *H. pylori* prevalence and predictor factors, age-standardized incidence rates were forecasted for each policy scenario from 2013 to 2050 (Fig. 4). 95% PIs overlapped across scenarios F1 (current policy scenario) to F4 (current policy + 2000 and 2013 policy effect) in 2030. However, the 95% PI for F1 (current policy scenario) did not overlap with that of F5 (current policy + 75% decrease) in 2030. Specifically, F1 (current policy scenario) demonstrated the highest incidence of 31.4 (95% PI 27.7–35.4), whereas F5 (current policy + 75% decrease) had the lowest incidence of 22.2 (95% PI 19.3–25.1) in 2030. Nevertheless, across all scenarios, age-standardized incidence rates plateaued, and the 95% PI overlapped at approximately 21–24 cases per 100,000 by 2050.

We forecasted gastric cancer incidence rates by sex for future policy scenarios (Supplementary Figs. S6 and S7). The pattern of incidence rates across scenarios differed between males and females. Specifically, for males, the 95% PI for F1 (current policy scenario): 41.8 (95% PI 36.5–47.7) did not overlap with those for F4 (current policy + 2000 and 2013 policy effect): 29.3 (95% PI 25.2–33.9) and F5 (current policy + 75% decrease): 24.2 (95% PI 20.6–28.2) in 2030. In contrast, for females, 95% PIs overlapped across all scenarios, F1-F5, in 2030. Nevertheless, for both males and females, the 95% PIs across all scenarios overlapped in 2050.

### Future prediction by different eradication target age

*H. pylori* prevalence and age-standardized incidence rates were predicted by age groups: 20–39 years old and 40–69 years old. Supplementary Figure S8 illustrates the *H. pylori* prevalence for the 20–39 age group across future policy scenarios F1–F5. The projected prevalence in 2030 was less than 10% in all policy scenarios: F1 (current policy scenario): 6.7%, F2 (current policy + 2000 policy effect): 5.0%, F3 (current policy + 2013 policy effect): 4.7%, F4 (current policy + 2000 and 2013 policy effect): 3.4%, and F5 (current policy + 75% decrease): 1.8%.

Supplementary Figure S9 illustrates the age-standardized gastric cancer incidence rates across future policy scenarios F1–F5 from 2013 to 2050 in the 20–39 age group. Notably, no substantial differences were observed across scenarios, with an overlap in the 95% PI across all future policy scenarios for the 20–39 age group. For example, the incidence for F1 (current policy scenario): 0.2 (95% PI 0.1–0.4) was similar to F5 (current policy + 75% decrease): 0.2 (95% PI 0.1–0.3) in 2030.

For the 40–69 age group (Supplementary Fig. S10), in comparison to F1 (current policy scenario), which projects a prevalence rate of 18.5% by 2030, other policy scenarios yield the following estimates: F2 (Current policy + 2000 policy effect): 13.9%; F3 (Current policy + 2013 policy effect): 12.3%, F4 (Current policy + 2000 and 2013 policy effect): 9.2%, and F5 (Current policy + 75% decrease): 4.6%.

Supplementary Figure S11 demonstrates the forecasted gastric cancer incidence rates across future policy scenarios F1–F5 in the 40–69 age group. 95% PI overlapped across scenarios F1 (current policy scenario), F2 (current policy + 2000 policy effect), and F3 (current policy + 2013 policy effect) in 2030. However, in 2030, F1 (current policy scenario) showed a lower incidence rate, with no overlap in the 95% PI: F1: 44.4 (95% PI 39.8–49.4), compared with F4 (current policy + 2000 and 2013 policy effect): 34.8 (95% PI 30.9–39.0) and F5 (Current policy + 75% decrease): 30.6 (95% PI 27.1–34.5). The incidence rates for the 40–69 age group plateaued at a higher level compared to the 20–39 age group, stabilizing at approximately 27–30 cases per 100,000 in 2050.

## Discussion

### Summary of findings

To the best of our knowledge, this is the first population-level study in Japan to evaluate the effect of *H. pylori* eradication policies on gastric cancer incidence. Our study demonstrated notable reductions in gastric cancer incidence rates due to eradication policies implemented in 2000 and 2013. Specifically, without these policy changes, higher gastric cancer rates were observed, underscoring the effectiveness of proactive eradication measures. In the future policy evaluation, continued *H. pylori* eradication was associated with further reductions in gastric cancer incidence, underscoring the importance of sustained eradication efforts as a key public health strategy.

This study demonstrated significant reductions in gastric cancer incidence rates due to *H. pylori* eradication policies implemented in 2000 and 2013. Specifically, in the absence of policy changes in 2000 and 2013, higher gastric cancer rates were observed compared to the current policy scenario, underscoring the effectiveness of expanding health insurance coverage for eradication measures.

Furthermore, projections for 2030 and 2050 suggest that expanding *H. pylori* eradication efforts could further reduce gastric cancer incidence, particularly when comparing the current policy scenario to a 75% increase in *H. pylori* eradication by 2030. Greater modeled impact in older adults likely reflects their higher baseline incidence, and early eradication remains critical for achieving long-term preventive benefits. Supporting this, Jung et al. (2023) found that among individuals with a family history of gastric cancer, *H. pylori* eradication before age 45 was associated with a 66% lower risk compared to those aged ≥ 75, whereas no significant difference was observed for the 70–74 age group [28]. Although there is no official age limit for *H. pylori* eradication, in older adults’ treatment, decisions should be individualized, weighing the benefits of cancer prevention against risk of side effects.

### Evaluation of past policies

Our findings underscore a substantial decrease in gastric cancer incidence in Japan with increasing *H. pylori* eradication policies, particularly within the 20–69 age group. Notably, P1 (no policy change in 2000 and 2013) exhibited a higher incidence of gastric cancer compared to P3 (current policy scenario) from 2017 onwards. This suggests the effectiveness of the Japanese government’s policies in approving *H. pylori* eradication therapy for individuals with peptic ulcers in 2000, and the subsequent expansion of this policy in 2013 to include all *H. pylori*-positive individuals.

That said, the observed overlap in the 95% PI between P3 (current policy scenario) and P2 (No policy change in 2013) could partially be attributed to the significant reduction in *H. pylori* prevalence already achieved by 2013, thus diminishing the potential impact of the new policy. From 2001 to 2013, approximately 600,000 individuals annually received *H. pylori* eradication therapy, with a total of 13.6 million people treated by 2016 [15, 19]. Following the policy expansion in 2013, the number of individuals receiving treatment increased by 1.4–1.6 million per year. In addition, by 2016, an estimated 40% of individuals aged 50–79 who tested positive for *H. pylori* had undergone eradication therapy [29]. This highlights the significance of both the 2000 and 2013 policies in effectively reducing the incidence of gastric cancer in Japan.

Although *H. pylori* infection rates have declined, this trend coincides with significant changes in eradication therapy—most notably, the introduction of vonoprazan in Japan, a potassium-competitive acid blocker. Vonoprazan-based triple therapy (vonoprazan, amoxicillin, and clarithromycin) achieves approximately 90% eradication rates, outperforming proton pump inhibitor (PPI)-based regimens [30]. Deguchi et al. further demonstrated that the overall success rate of primary eradication therapy increased following the introduction of vonoprazan in 2015 [31]. While improved treatment efficacy likely contributed to better individual outcomes, it does not fully account for the broader declines in *H. pylori* prevalence and gastric cancer incidence. Such trends may reflect a combination of enhanced therapeutic effectiveness and national policy initiatives.

### Future prediction by different eradication effects

We aimed to evaluate the potential impact of expanding *H. pylori* eradication on future gastric cancer incidence rates in the 20–69 age group, specifically considering scenarios with the 2000 policy effect, the combined 2000 and 2013 policy effects, and a 75% decrease in *H. pylori* prevalence by 2030 (Figs. 3 and 4).

Our findings indicate that in 2030, the 95% PI for F4 (current policy + 2000 and 2013 policy effect) and F5 (current policy + 75% decrease) did not overlap with F1 (current policy scenario), suggesting significant potential reductions in gastric cancer incidence with increased eradication efforts. This reduction in *H. pylori* prevalence suggests significant potential for further decreases in both *H. pylori* prevalence and gastric cancer incidence. In addition, other studies support that increased *H. pylori* eradication can lead to decreased gastric cancer incidence, even at low prevalence rates around 5% [19, 32].

However, by 2050, incidence rates across these scenarios converged, indicating that the initial differences in policy impacts diminished over time, likely due to the widespread reduction in *H. pylori* prevalence and other long-term health improvements.

### Future prediction by different eradication target age

It is well established that the prevalence of *H. pylori* infection is considerably higher in older adults compared to younger adults. This disparity can be attributed to increased exposure to contaminated water supply systems before the rapid economic growth and urbanization of the 1960 s and 1970 s [7, 33]. During this period, inadequate water sanitation led to a higher incidence of *H. pylori* infections among individuals who were children or young adults at the time. Consequently, the overall prevalence of *H. pylori* has decreased among younger generations, who have benefited from improved water supply systems and better living conditions, thereby reducing their risk of exposure [17, 34].

However, differences in infection prevalence alone do not fully account for the greater effectiveness of eradication policies observed in older age groups. Evidence from Japan suggests that the duration of *H. pylori* infection and the presence of precancerous gastric changes at the time of eradication are critical determinants of cancer prevention efficacy. A meta-analysis reported an odds ratio (OR) of 0.39 (95% CI 0.31–0.49) for gastric cancer prevention following eradication, with even greater benefit among individuals with benign gastric conditions (OR 0.32, 95% CI 0.19–0.54) [35]. Similarly, another systematic review demonstrated a reduction in gastric cancer risk among healthy individuals (OR 0.34, 95% CI 0.25–0.46), although the protective effect was attenuated in those with established precancerous lesions [36]. Modeling studies further indicate that the lifetime risk of gastric cancer is markedly higher in infected compared to uninfected individuals, and that eradication is most effective when implemented before the onset of significant mucosal damage [37]. In addition, the duration of *H. pylori* infection plays a critical role in gastric cancer development. Previous studies have demonstrated that histopathological scores worsen by approximately 0.20 units for each year of persistent infection [38]. Collectively, these findings emphasize that the timing of eradication and the underlying gastric mucosal status also play a pivotal role in determining the success of prevention strategies, in addition to differences in infection prevalence.

Furthermore, while our model indicates that the most substantial short-term reductions in gastric cancer incidence occur among adults aged 40–69, reflecting their higher baseline prevalence, this does not diminish the importance of early eradication. Notably, our model’s time horizon, limited to 2050, may underestimate the long-term protective effects of early eradication emphasized in current clinical guidelines. Our findings highlight that early eradication at younger ages may confer delayed but clinically meaningful benefits by interrupting the progression to atrophic gastritis and intestinal metaplasia [39]. Previous modeling studies have evaluated the optimal age for *H. pylori* screening and treatment. A state-transition model of individuals aged 15–80 years identified age 15 as the most cost-effective age for *H. pylori* screening and treatment in Japan [32]. Similarly, a microsimulation model calibrated to SEER gastric cancer incidence and natural history data from clinical studies found that a screen-and-treat strategy provided the greatest population-level benefit when implemented before age 40 in the US [40]. Although the impact of *H. pylori* detection and eradication on gastric cancer may become more apparent later in life, when cancer prevalence is highest, early intervention may remain essential to achieve these long-term population-level benefits.

In addition, with the widespread adoption of *H. pylori* eradication therapy and improvements in public health measures, there is an increasing need to focus on gastric cancers arising in *H. pylori*-negative or previously eradicated individuals. Prior studies have reported that the proportion of *H. pylori*-negative gastric cancer ranges from 0.4% to 10.6% [41–44]. In addition, individuals with a family history of gastric cancer, hereditary cancer syndromes (e.g., CDH1 mutation carriers), or premalignant gastric lesions could be considered for genetic counseling and testing [39, 45]. In cases involving hereditary diffuse gastric cancer or other cancer-predisposing mutations (e.g., CDH1, BRCA1/2), prophylactic gastrectomy may be considered for select high-risk individuals based on clinical and genetic risk assessment [45, 46]. Although the absolute risk of gastric cancer is reduced in *H. pylori*-negative or post-eradication individuals, the European Society of Gastrointestinal Endoscopy recommends endoscopic surveillance every 3 years for patients with severe atrophic gastritis, gastric intestinal metaplasia involving both the antrum and body, or those classified as OLGA/OLGIM stage III/IV, irrespective of *H. pylori* status [47, 48]. Notably, gastric cancer risk differs significantly between individuals who were never infected and those previously infected, even after eradication [49, 50]. Therefore, regular endoscopic surveillance remains warranted in individuals with advanced histologic alterations following eradication, as these patients continue to exhibit an elevated risk of neoplastic progression.

As the epidemiology of gastric cancer continues to evolve, further research will be necessary to parse infection status and monitor trends in *H. pylori*-negative gastric cancer incidence for developing targeted intervention strategies.

### Additional strategies to decrease gastric cancer incidence

As previously mentioned, the incidence rates of gastric cancer across policy scenarios are projected to converge by 2050, indicating diminishing returns from *H. pylori* eradication efforts over time. Although *H. pylori* accounts for approximately 80–90% of gastric cancer cases, other lifestyle factors may have contributed to recent trends [51, 52]. In 2015, the population attributable fractions for gastric cancer in Japan were estimated at 21.5% for smoking, 5.6% for alcohol consumption, 0.4% for body mass index, 17.7% for high-salt food intake, and 1.8% for low vegetable consumption [53].

In Japan, there has been a Shift in dietary patterns toward a more Westernized diet. This dietary transition may have contributed, in part, to the steady decline in sodium intake among Japanese adults, who have traditionally consumed approximately 13.9 g of salt per day, compared to 3.5 g per day in the United States [54, 55]. However, the reduction in salt intake cannot be attributed solely to dietary Westernization, as public health initiatives and advancements in food processing and preservation have also been instrumental [56, 57]. In addition, increased consumption of red and processed meats and saturated fats, hallmarks of Western dietary patterns, has been associated with higher risks of other cancers and metabolic diseases, potentially offsetting some of the health gains achieved [58, 59].

In addition to dietary changes, reductions in smoking prevalence have also contributed to the decline in gastric cancer incidence. According to a study by Tanaka et al., the overall smoking prevalence among men aged 25–64 years decreased from 56.0% in 2001 to 38.4% in 2016, and among women from 17.0% to 13.0% during the same period [60]. In 2019, DALYs attributable to smoking for gastric cancer were estimated at 23% in Japan [61]. However, socioeconomic disparities in smoking behavior have widened, with higher smoking rates persisting among lower socioeconomic groups [60].

Similarly, alcohol consumption in Japan has steadily declined in recent years [62]. Previous studies have demonstrated a positive association between higher levels of alcohol intake and increased risk of gastric cancer [63]. A pooled analysis of six cohort studies reported hazard ratios (HRs) rising from 1.09 for 23 to < 46 g/day to 1.29 for ≥ 92 g/day in men [64]. Nonetheless, according to the Ministry of Health, Labour, and Welfare, as of 2022, 13.5% of Japanese men and 9.0% of women continue to consume alcohol at levels associated with an elevated risk of lifestyle-related diseases, indicating that further reductions remain necessary [65].

Addressing these risk factors is crucial for reducing gastric cancer incidence in Japan. Japan has implemented several initiatives to reduce population-wide salt intake, with the Health Japan 21 program setting a target of reducing average daily salt consumption to 7 g by 2032 [66]. Potential strategies to support this goal include front-of-pack nutrition labeling, reformulation of processed foods to reduce sodium content, and cross-sectoral collaborations to encourage healthier dietary practices [55]. In addition, while reductions in smoking and alcohol consumption have yielded some success, there remains a high prevalence of these behaviors, especially among men [67, 68]. Policies such as increased taxation on tobacco and alcohol, comprehensive cessation programs, and community- and workplace-based interventions may help address these risk factors and contribute to reducing gastric cancer incidence [69–71].

### Strength and limitations

Our study has several strengths. First, our study includes the use of representative, population-based data, considering not only the effects of *H. pylori* eradication policies on age-standardized gastric cancer incidence rates but also confounding factors such as smoking, alcohol consumption, salt intake, and BMI. Second, the models were validated, adding to the credibility of our findings. Moreover, the scenarios analyzed in this study were based on real-world estimates from previous literature, thereby enhancing the relevance of the results regarding the impact of these policy changes. Third, the integration of historical prevalence data with long-term forecasts provides a comprehensive evaluation of policy impacts over time, enhancing the study’s relevance for future public health planning.

However, our study has several limitations. First, we were missing alcohol consumption data for some age groups, which could potentially influence the results. Second, we did not employ a dynamic model due to our understanding that new infections of *Helicobacter pylori* are frequent. Third, our study indirectly measures the effect of eradication efforts through observed decreases in *H. pylori* infection rates, based on historical data and previous studies. However, this approach may not fully capture the complexity of eradication dynamics and other influencing factors. Fourth, our analysis relied on sex-specific smoking prevalence data but lacked comparable sex-specific *H. pylori* prevalence rates, potentially impacting the interpretation and generalizability of our findings. Fifth, although older adults represent a significant portion of the gastric cancer burden, individuals aged ≥ 70 were excluded due to the limited availability of high-quality, age-specific predictor data (e.g., smoking status) and the potential variable effectiveness of *H. pylori* eradication in the elderly, both of which introduced uncertainty in modeling this population [28]. Collectively, these factors constrained our ability to reliably incorporate this age group within the current analytic framework. Sixth, because insurance coverage began in 2000, it is challenging to evaluate the impact of coverage in the absence of a longer historical trend. However, extending the study period further back is not feasible due to the unavailability of high-quality, comprehensive population-based risk and health predictor data prior to 1990. Finally, there was a moderate underestimation of gastric cancer incidence in the 20–39 age group, which may have resulted in a diminished estimate of the potential impact of *H. pylori* eradication on reducing incidence in this population.

In conclusion, the *H. pylori* eradication policies implemented in 2000 and 2013 have markedly reduced age-standardized gastric cancer incidence rates in Japan, and further reductions are likely achievable through continued and expanded eradication efforts. While our findings indicate the greatest declines in incidence occurred among adults aged 40–69 years, it highlights that the benefits of early *H. pylori* detection and eradication may be more evident later in life, when gastric cancer rates are the highest. Consistent with current Japanese guidelines, it remains essential to ensure access to eradication therapy, particularly before the development of atrophic gastritis or intestinal metaplasia, as a cornerstone of long-term gastric cancer prevention.

## Supplementary Information

Below is the link to the electronic supplementary material.

Supplementary file 1 (DOCX 27 kb)

Supplementary file 2 (EPS 9 kb)

Supplementary file 3 (EPS 9 kb)

Supplementary file 4 (EPS 9 kb)

Supplementary file 5 (EPS 16 kb)

Supplementary file 6 (EPS 15 kb)

Supplementary file 7 (EPS 17 kb)

Supplementary file 8 (EPS 22 kb)

Supplementary file 9 (EPS 12 kb)

Supplementary file 10 (EPS 18 kb)

Supplementary file 11 (EPS 12 kb)

Supplementary file 12 (EPS 22 kb)

## Abbreviations

ARIMA: Autoregressive integrated moving average model
ARIMAX: Autoregressive integrated moving average with exogenous variables
KPSS test: Kwiatkowski–Phillips–Schmidt–Shin test
