# Supplementary material for: Forecasting age-standardized incidence rates of gastric cancer from 1990–2050 in Japan according to H. pylori prevalence and eradication scenarios
Source: J Gastroenterol. 2025 Sep 19;60(11):1372–83. doi: 10.1007/s00535-025-02296-y (PMC12549766; doi:10.1007/s00535-025-02296-y)
Supplement: Supplementary file 1 — (DOCX 27 kb) [file 535_2025_2296_MOESM1_ESM.docx]

**Supplementary Document**

**Supplementary Figure 1**. Validation of ARIMAX model for age-standardized gastric cancer incidence among individuals aged 20–69 years, both sexes, from 1990 to 2015.

Observed incidence rates with model-estimated values to evaluate model fit among 20-69 age group.

**Supplementary Figure 2**. Validation of ARIMAX model for age-standardized gastric cancer incidence among individuals aged 20–39 years, both sexes, from 1990 to 2015.

Observed incidence rates with model-estimated values to evaluate model fit among 20-39 age group.

**Supplementary Figure 3**. Validation of ARIMAX model for age-standardized gastric cancer incidence among individuals aged 40–69 years, both sexes, from 1990 to 2015.

Observed incidence rates with model-estimated values to evaluate model fit among 40-69 age group.

**Supplementary Figure 4**. Projected age-standardized gastric cancer incidence among males aged 20–69 years from 1990 to 2050 under three alternative past policy scenarios: P1 (no policy change in 2000 and 2013); P2 (no policy change in 2013); P3 (current policy scenario).

More extensive *H. pylori* eradication policies were associated with greater projected reductions in gastric cancer incidence among males.

**Supplementary Figure 5**. Projected age-standardized gastric cancer incidence among females aged 20–69 years from 1990 to 2050 under three alternative past policy scenarios: P1 (no policy change in 2000 and 2013); P2 (no policy change in 2013); P3 (current policy scenario).

The implementation of additional *H. pylori* eradication policies is associated with larger projected reductions in gastric cancer incidence among females.

**Supplementary Figure 6**. Projected age-standardized gastric cancer incidence rate among males aged 20–69 years from 1990 to 2050 under five alternative future policy scenarios: F1 (current policy scenario); F2 (current policy + 2000 policy effect); F3 (current policy + 2013 policy effect); F4 (current policy + 2000 and 2013 policy effects); F5 (current policy + 75% decrease).

More intensive future public health interventions are associated with greater projected reductions in gastric cancer incidence among males.

**Supplementary Figure 7**. Projected age-standardized gastric cancer incidence rate

among females aged 20–69 years from 1990 to 2050 under five alternative future policy scenarios: F1 (current policy scenario); F2 (current policy + 2000 policy effect); F3 (current policy + 2013 policy effect); F4 (current policy + 2000 and 2013 policy effects); F5 (current policy + 75% decrease).

95% prediction intervals overlapped across scenarios, indicating similar additional benefit of expanded policies on gastric cancer incidence among females by 2030.

**Supplementary Figure 8**. Projected *H. pylori* prevalence among individuals aged 20–39 years, both sexes, from 1990 to 2050 under five alternative future policy scenarios: F1 (current policy scenario); F2 (current policy + 2000 policy effect); F3 (current policy + 2013 policy effect); F4 (current policy + 2000 and 2013 policy effects); F5 (current policy + 75% decrease).

Decreased projected *H. pylori* prevalence across all scenarios by 2030, with lower variation due to the already low baseline prevalence in 20-39 age group.

**Supplementary Figure 9**. Projected age-standardized gastric cancer incidence rate among individuals aged 20–39 years, both sexes, from 1990 to 2050 under five alternative future policy scenarios: F1 (current policy scenario); F2 (current policy + 2000 policy effect); F3 (current policy + 2013 policy effect); F4 (current policy + 2000 and 2013 policy effects); F5 (current policy + 75% decrease).

Similar projected incidence across scenarios, with substantial overlap in 95% prediction intervals through 2030, reflecting similar added benefit of expanded eradication efforts in 20-39 age group.

**Supplementary Figure 10**. Projected *H. pylori* prevalence among individuals aged 40–69 years, both sexes, from 1990 to 2050 under five alternative future policy scenarios: F1 (current policy scenario); F2 (current policy + 2000 policy effect); F3 (current policy + 2013 policy effect); F4 (current policy + 2000 and 2013 policy effects); F5 (current policy + 75% decrease).

Substantial variation in projected *H. pylori* prevalence by 2030 in 40-69 age group, with the greatest reductions observed under more intensive eradication scenarios.

**Supplementary Figure 11**. Projected age-standardized gastric cancer incidence rate among individuals aged 40–69 years, both sexes, from 1990 to 2050 under five alternative future policy scenarios: F1 (current policy scenario); F2 (current policy + 2000 policy effect); F3 (current policy + 2013 policy effect); F4 (current policy + 2000 and 2013 policy effects); F5 (current policy + 75% decrease).

Expanded eradication efforts are associated with greater projected reductions in gastric cancer incidence in 40-69 age group, with F4 and F5 demonstrating non-overlapping 95% prediction intervals compared to F1 by 2030.
